# Synthesis and Cytotoxicity of Silicon Containing Pyridine and Quinoline Sulfides

**DOI:** 10.1155/MBD.2002.45

**Published:** 2002

**Authors:** Edmunds Lukevics, Edgars Abele, Pavel Arsenyan, Ramona Abele, Kira Rubina, Irina Shestakova, Ilona Domracheva, Violetta Vologdina

**Affiliations:** 1 Latvian Institute of Organic Synthesis 21 Aizkraukles Street Riga LV-I006 Latvia

## Abstract

Silicon containing pyridine and quinoline sulfides have been prepared using phase transfer catalytic system thiol/alkyl halide / solid KOH/18-crown-6 / toluene. The target S-ethers were isolated in yields up to 81%. The cytotoxicity of the synthesized compounds was studied. Among pyridine sulfides S-[3-(1-methyl- 1-silacyclohexyl)propyl] derivatives **5e** and **6e** exhibit the highest cytotoxicity. Aliphatic silicon derivatives were considerably less active. 8-[(Trimethylsilylmethyl)thio]quinoline (**8a**) exhibits the highest activity among quinoline sulfides.


### Synthesis and Cytotoxicity of Silicon Containing Pyridine and Quinoline Sulfides

Edmunds Lukevics, Edgars Abele, Pavel Arsenyan, Ramona Abele, Kira Rubina, Irina Shestakova, Ilona Domracheva, and Violetta Vologdina

*Latvian Institute of Organic Synthesis*
*21 Aizkraukles Street, Riga, LV-1006, Latvia*
*E-mail: kira@osi.lv*

### ABSTRACT

Silicon containing pyridine and quinoline sulfides have been prepared using phase transfer catalytic system thiol /alkyl halide / solid KOH/18-crown-6 / toluene. The target S-ethers were isolated in yields up to 81%. The cytotoxicity of the synthesized compounds was studied. Among pyridine sulfides S-[3-(1-methyl-1-silacyclohexyl)propyl] derivatives **5e** and **6e** exhibit the highest cytotoxicity. Aliphatic silicon derivatives were considerably less active. 8-[(Trimethylsilylmethyl)thio]quinoline **(8a)** exhibits the highest activity among quinoline sulfides.

### INTRODUCTION

Pyridine and quinoline sulfides and related compounds exhibit a wide range of biological activity /1/. Among these activities antitumor and cytotoxic activities of pyridine /2-8/ and quinoline /9-12/ sulfides were described.

The known methods for the preparation of sulfides are based on reaction of hetaryl thiols with alkyl or aryl halides in the presence of $K_2CO_3$ / $Me_2Co$ /13/, NaOMe / DMF /14/ or NaH / $Me_2SO_4$ /15/ systems. Recently we described two simple phase transfer catalytic (PTC) methods for the preparation of hetaryl sulfides in the hetaryl thiol / alkyl halide / solid $K_2CO_3$ / 18-crown-6 / toluene /1/ or hetaryl S-acetate / alkyl halide / solid KOH / 18-crown-6 / benzene systems /16/.

We have found that 3-(hataryltio)-1-propynyl(trimethyl)silanes exhibit high cytotoxicity /17/. In the present work the novel N-beterocyclic sulfides with trialkylsily and silacyclic substituents have been synthesized as potential antitumor agents.

### MATERIALS AND METHODS

### Chemistry

$^1H$ NMR spectra were recorded on a Varian 200 Mercury instrument using $CDCl_3$ as a solvent and hexamethyldisiloxane (HMDSO) as an internal standard (0.055 ppm). Mass spectra were registered on a GC-MS HP 6890 (70 eV). GC analysis was performed on a Chrom-5 instrument equipped with flame-ionization detector using glass column packed with 5% OV-101 / Chromosorb W-HP (80-100 mesh) (1.2 m × 3 mm). Bromomethyltrimethylsilane, 3-iodopropyltrimethylsilane, 1-(3-iodopropyl)-1-methylsilacyclopentane and 1-(-iodopropyl)-1-methylsilacyclohexane were obtained by Grignard reaction /18,19/ from corresponding chloropropylmethyldichlorosilane with the following exchange of chlorine atom by iodine using $NaI/(CH_3)_2CO$ in excellent yields.

***General procedure for alkylation of thiols* 1-4.**

Finely powdered dry $K_2CO_3$ (0.82 g, 6 mmol) was added to a suspension of thiol **1-4** (compound **4** was used as potassium salt) (2 mmol), silane (2 mmol) and 18-crown-6 (0.053 g, 0.2 mmol) in 1.5 ml of toluene. The mixture was refluxed with stirring to achieve the disappearance of the substrates, filtered over the thin silica gel layer and concentrated under reduced pressure. The residue was purified by column chromatography on silicagel (eluent benzene – ethyl acetate in different mixtures) to give products **5-8**. The results are shown in Tables 1 – 3.

### In vitro cycotoxicity assay

Monolayer cell lines were cultivated for 72 h in DMEM standard medium without an indicator and antibiotics. After the ampoule was defrozen not more than four passages were performed. The control cells and cells with tested substances in the range of $2\text{-}5 \cdot 10^4$ cell/mL concentration (depending on line nature) were placed on separate 96 wells plates. Solutions containing test compounds were diluted and added in wells to give the final concentrations of 50, 25, 12.5, and 6.25 μg/mL. Control cells were treated in the same manner only in the absence of test compounds. Plates were cultivated for 72 h. The quantity of survived cells was determined using crystal violet (CV) or 3-(4,5-dimethylthiazol-2-yl)-2,5-diphenyltetrazolinium bromide (MTT) coloration that was assayed by multiscan spectrophotometer. The quantity of alive cells on the control plate was taken in calculations as 100% /20,21/. The concentration of NO was determined according to /20/.

### RESULTS AND DISCUSSION

### Chemistry

Alkylation of pyridine and quinoline thiols **1-4** has been carried out in the phase transfer catalytic system silyl alkyl halide / solid $K_2CO_3$ /18-crown-6 / toluene at reflux. Sulfides **5-8** were isolated in 20-81% yield by column chromatography (Table 1).

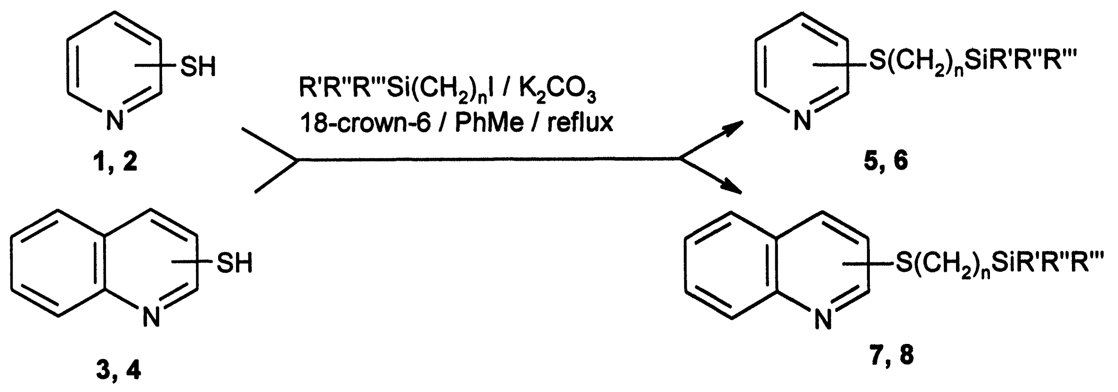

The spectroscopic data of compounds **5c**, **6b**, **6c**, **6e**, **7b**, **7c**, **7e** are presented in Tables 2, 3. Compounds **5b**, **5e**, **8a**, **8d**, **8e** were described in /1/.

**TABLE 1**

Synthesis of silyl derivatives of hetaryl thiols

| Thiol | Het | SiR'R"R'" | n | Reaction time, h | Product | Isolated yield, % |
|---|---|---|---|---|---|---|
| **1** | 2-pyridyl | $SiMe_3$ | 3 | 8 | **5b** | 66 |
| **1** | 2-pyridyl | $SiMe_2Hp$ | 3 | 7 | **5c** | 36 |
| **1** | 2-pyridyl | 1-Me-1- silacyclohexyl | 3 | 9 | **5e** | 60 |
| **2** | 4-pyridyl | $SiMe_3$ | 3 | 7 | **6b** | 62 |
| **2** | 4-pyridyl | $SiMe_2Hp$ | 3 | 7 | **6c** | 32 |
| **2** | 4-pyridyl | 1-Me-1- silacyclohexyl | 3 | 7 | **6e** | 38 |
| **3** | 2-quinolyl | $SiMe_3$ | 3 | 7 | **7b** | 53 |
| **3** | 2-quinolyl | $SiMe_2Hp$ | 3 | 7 | **7c** | 57 |
| **3** | 2-quinolyl | 1-Me-1- silacyclohexyl | 3 | 7 | **7e** | 64 |
| **4** | 8-quinolyl | $SiMe_3$ | 1 | 7 | **8a** | 45 |
| **4** | 8-quinolyl | 1-Me1- silacyclopentyl | 3 | 21 | **8d** | 20 |
| **4** | 8-quinolyl | 1-Me-1- silacyclohexyl | 3 | 9 | **8e** | 24 |

**TABLE 2**

$^1H$ NMR data of pyridine and quinoline sulfides

| Compound | δ (ppm, $CDCl_3$ / HMDSO) |
|---|---|
| **5c** | -0.01(s, 6H, $SiCH_3$), 0.51 (m, 2H, $SiC\underline{H}_2(CH_2)_5CH_3$), 0.68 (m,2H, $SiCH_2$), 0.8-1.7 (m, 15H, $CH_2C\underline{H}_2CH_2$ and $CH_2(C\underline{H}_2)_5C\underline{H}_3$), 3.18 (t, 2H, J = 7.4 Hz, $CCH_2$), 6.98 (m, 1H, 5-H), 7.17 (m, 1H, 3-H), 7.44(m, 1H, 4-H), 8.45 (m, 1H, 6-H) |
| **6b** | 0.00 (s, 9H, $Si(CH_3)_3$), 0.66 (m, 2H, $CH_2Si$), 1.70 (m, 2H, $CH_2C\underline{H}_2CH_2Si$), 2.97 (t, 2H, J= 7.2 Hz, $SCH_2$), 7.09 (m, 1H, 3H and 5-H), 8.37 (m, 1H, 2-H and 6-H), |
| **6c** | -0.03(s, 6H, $SiCH_3$), 0.49 (m, 2H, $SiC\underline{H}_2(CH_2)_5CH_3$), 0.66 (m,2H, $SiCH_2$), 0.8-1.7 (m, 15H, $CH_2C\underline{H}_2CH_2$ and $CH_2(C\underline{H}_2)_5C\underline{H}_3$), 2.97 (t, 2H, J = 7.4 Hz, $CCH_2$), 7.09 (m, 2H, 3-H and 5-H), 8.37 (m, 2H, 2-H and 6-H) |
| **6e** | 0.05 (s, 3H, $SiCH_3$), 0.60 (m, 6H, $SiCH_2$), 1.63 (m, 8H, $CH_2(C\underline{H}_2)_3CH_2$ in silacycle and $CH_2C\underline{H}_2CH_2Si$), 2.97 (t, 2H, J= 7.4 Hz, $SCH_2$), 7.09 (m, 2H, 3-H and 5-H), 8.37 (m, 2H, 2-H and 6-H) |
| **7b** | 0.02 (s, 9H, $Si(CH_3)_3$), 0.76 (m, 2H, $CH_2Si$), 1.80 (m, 2H, $CH_2C\underline{H}_2CH_2Si$), 3.34 (t, 2H, J= 7.4 Hz, $SCH_2$), 7.22, 7.44, 7.67 and 7.90 (all m, 6H, quinoline ring protons) |
| **7c** | -0.02(s, 6H, $SiCH_3$), 0.51 (m, 2H, $SiC\underline{H}_2(CH_2)_5CH_3$), 0.72 (m,2H, $SiCH_2$), 0.9-1.8 (m, 15H, $CH_2C\underline{H}_2CH_2$ and $CH_2(C\underline{H}_2)_5C\underline{H}_3$), 3.34 (t, 2H, J = 7.4 Hz, $CCH_2$), 7.23, 7.40, 7.68 and 7.90 (all m, 6H, quinoline ring protons) |
| **7e** | 0.05 (s, 3H, $SiCH_3$), 0.57 and 0.67 (both m, 6H, $SiCH_2$), 1.64 (m, 8H, $CH_2(C\underline{H}_2)_3CH_2$ in silacycle and $CH_2C\underline{H}_2CH_2Si$), 3.36(t, 2H, J= 7.0 Hz, $SCH_2$), 7.43, 7.69, 7.90 and 8.00 (all m, 6H, quinoline ring protons) |

**TABLE 3**

Mass-spectroscopic data of pyridine and quinoline sulfides

| Compound | m/z (intensity, %) |
|---|---|
| **5c** | 294 ($M^+$- Me, 10), 262 (5), 210 (100), 168 (52), 154 (7), 138 (13), 111 (53), 78 (17), 59 (38) |
| **6b** | 225 ($M^+$, 5), 210 (97), 183 (9), 168 (57), 151 (7), 73 (100), 59 (14), 51 (15), 45 (23), 39 (13) |
| **6c** | 308 ($M^+$-1, <1), 294 (5), 210 (100), 168 (42), 154 (7), 138 (4), 73 (5), 59 (31), 43 (7) |
| **6e** | 265 ($M^+$, 26), 222 (100), 209 (12), 195 (12), 180 (41), 166 (28), 152 (15), 138 (10), 113 (27), 85 (42), 59 (17), 51 (16), 43 (20) |
| **7b** | 275 ($M^+$, 3), 260 (8), 228 (8), 218 (18), 188 (15), 175 (21), 161 (100), 128 (38), 101 (12), 73 (33), 45 (12) |
| **7c** | 360 ($M^+$, 1), 344 (4), 312 (5), 260 (37), 218 (33), 188 (11), 175 (13), 161 (100), 143 (12), 128 (29), 59 (36) |
| **7e** | 315 (22), 272 (83), 244 (28), 231 (38), 217 (33), 188 (18), 174 (13), 161 (100), 143 (13), 128 (67), 101 (14), 85 (32), 59 (21), 43 (20) |

### *In vitro* cytotoxicity

Cytotoxic activity of synthesized silicon-containing sulfides **5-8** was tested *in vitro* on two monolayer tumor cell lines: MG-22A (mouse hepatoma) and HT-1080 (human fibrosarcoma). Concentrations providing 50% of tumor death effect were determined according to the known procedure /22/ using 96 well plates.

The experimental evaluations of cytotoxic properties are presented in Table 4. A preliminary analysis of the structure-activity relationship for the cytotoxic action clearly indicates the strong influence of the silylalkyl substituent structure.

**TABLE 4**

*In vitro* cell cytotoxicity and the ability of intracellular NO generation caused by silicon and containing pyridine and quinoline sulfides

| N° | Compound | HT- 1080 | | MG- 22A | |
|---|---|---|---|---|---|
| | | $TD_{50}$[a] | NO, % CV[b] | $TD_{50}$[a] | NO, % CV[b] |
| **5b** |  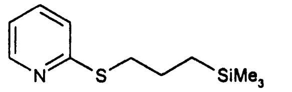 | 4 | 400 | 4 | 300 |
| **5c** |  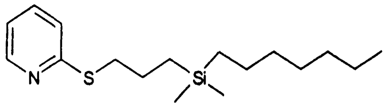 | 78 | 44 | 39 | 650 |
| **5e** |  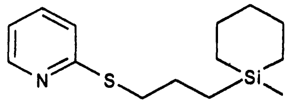 | 6.5 | 500 | 4.5 | 400 |
| **6b** |  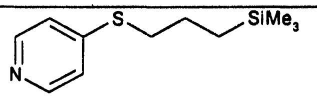 | 67 | 167 | 1.2 | 250 |
| **6c** |  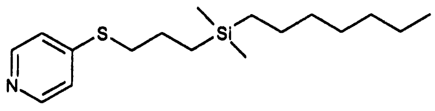 | 52 | 114 | 15.5 | 275 |
| **6e** |  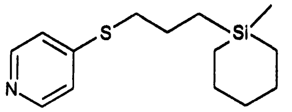 | 3 | 500 | <1 | 450 |
| **7b** |  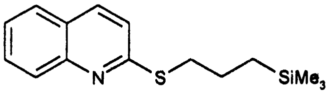 | 73 | 250 | 3.8 | 200 |
| **7c** |  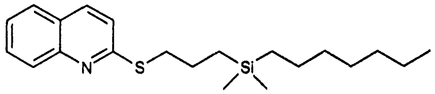 | >100 | 33 | >100 | 36 |
| **7e** |  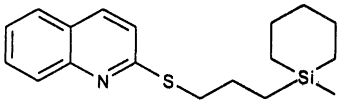 | 47 | 30 | 34 | 157 |
| **8a** |  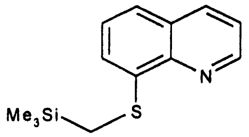 | 2.5 | 350 | 3.5 | 200 |
| **8d** |  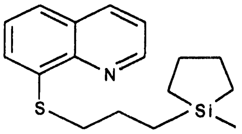 | 17 | 300 | 22 | 200 |
| **8e** |  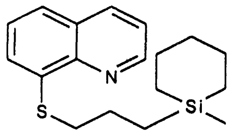 | 21.5 | 350 | 8.5 | 400 |

[a] Concentration (μg/mL) providing 50% cell killing effect [(CV+MTT)/2)

[b] NO concentration (%) (CV: coloration).

Pyridine and quinoline sulfides bearing dimethylheptylsilyl group at the sulfur atom (**5c**, **6c**, and **7c**) have a slight cytotoxic effect (> 15.5 µg/mL). The substitution of dimethylheptylsilyl group by trimethylsilyl (**5b**, **6b**, and **7b**) or silahexyl group (**5e**, **6e**, and **7e**) results in considerable increase of the cytotoxic activity. It must be noted that the activity of studied compounds depends on the tumor type. In general, all silicon containing sulfides (**5-8**) show the expressed selectivity on mouse hepatoma MG – 22A cell line. However, 8-trimethylsilylmethylmercaptoquinoline **8a** exhibits a greater toxicity on HT – 1080 cells (2.5 µg/mL) contrary to MG – 22A (3.5 µg/mL). Comparison of the tumor growth inhibition for derivatives **5 – 7b** and **5 – 7 c** shows a higher cytotoxic activity of the trimethylsilylpropyl group containing sulfides with respect to dimethylheptylsilylpropyl substituted sulfides. Among pyridine derivatives 4-[3-(1-methyl-1-silacyclohexyl)propyl]pyridine sulfide **6e** exhibits the highest cytotoxicity on MG – 22A (<1 µg/mL). The most active in the series of quinoline sulfides is 8-[(trimethylsilylmethyl)thio]quinoline **8a** (2.5 µg/mL on human fibrosarcoma HT – 1080 cell line). Studied pyridine and quinoline derivatives have a medium NO-induction ability, 2-(3′-dimethylheptylsilylpropyl)pyridine sulfide **5c** being the most active (650% on MG – 22A test).

### REFERENCES

1. E. Abele, K. Rubina, R. Abele, I. Sleiksha and E. Lukevics, *Chem. Heterocyclic Comp.*, **35**, 1052 (1999).
2. T. Takahashi, K. Ueda and T. Ichimoto, *Chem. Pharm. Bull.*, **3**, 356 (1955); *Chem. Abstr.*, **50**, 16770b (1956).
3. L. Katz, M.S. Cohen and W. Schroeder, US Pat. 2824876 (1958); *Chem. Abstr.*, **52**, 12930c (1958).
4. J.L. Greene, Jr., A.M. Williams and J.A. Montgomery, *J. Med. Chem.*, **7**, 20 (1964).
5. W.C.J. Ross, *J. Med. Chem.*, **10**, 257 (1967).
6. M.J. Gil, M.A. Manu, C. Arteaga, M. Migliaccio, I. Encio, A. Gonzalez and V. Martinez-Merino, *Bioorg. Med. Chem. Lett.*, **9**, 2321 (1999).
7. A. Gangjee, Y. Zhu and S.F. Queener, *J. Med. Chem.*, **41**, 4533 (1998).
8. A.P. Krapcho, S.N. Haydar, S. Truong-Chiott, M.P. Hacker, E. Menta and G. Beggiolin, *Bioorg. Med. Chem. Lett.*, **10**, 305 (2000).
9. L. Monti, G. Granchi and C. Pellerano, *Gazz. Chim. Ital.*, **91**, 115 (1961).
10. W.O. Foye, Y.H. Kim and J.M. Kauffman, *J. Pharm. Sci.*, **72**, 1356 (1983).
11. W.O. Foye, S.H. An and T.J. Mayer, *J. Pharm. Sci.*, **73**, 1168 (1984).
12. D. Bergen, R. Citarellia, M. Dutia, L. Greenberger, W. Hallett, R. Paul and D. Powell, *J. Med. Chem.*, **42**, 2145 (1999).
13. J. Ehrenfreund, Eur. Pat. 22748 (1981); *Chem. Abstr.*, **95**, 7078b (1981).
14. V. Klimesova, M. Svoboda, K. Waisser, J. Kaustova, V. Buchta and K. Kralova, *Eur. J. Med. Chem.*, **34**, 433 (1999).

15. G. Scheffler, J. Engel, V. Jakovlev, B. Nickel and K. Thiemer, Eur. Pat. 149088 (1985); *Chem. Abstr.*, **103**, 215189z (1985).
16. E. Abele, R. Abele, J. Popelis and E. Lukevics, *Latv. J. Chem.*, **N2**, 61 (1998).
17. R. Abele, E. Abele, K. Rubina, O. Dzenitis, P. Arsenyan, I. Shestakova, A. Nesterova, I. Domracheva, J. Popelis, S. Grinberga and E. Lukevics, *Khim. Geterotsilk. Soedin,*, **2002**, 977.
18. N.S. Nametkin, K.S. Vdovin, K.S. Pushchevaya and V.I. Zawyalov, *Izv. Akad. Nauk SSSR, Otd. Khim. Nauk*, **1965**, 1453.
19. J.W. Wilt and C.F. Dockus, *J. Amer. Chem. Soc.*, **92**, 5813 (1970).
20. D.J. Fast, R.C. Lynch and R.W. Leu, *J. Leuckocyt. Biol.*, **52**, 255 (1992).
21. P.J. Freshney, *Culture of Animals Cells* (*A Manual of Basic Technique*), Wiley-Liss, New York, 1994; pp. 296-297.
22. R.J. Riddell, R.H. Clothier and M. Balls, *Fd. Chem. Toxicol.*, **24**, 469 (1986).

